# Evaluation of a machine learning algorithms for predicting the dental age of adolescent based on different preprocessing methods

**DOI:** 10.3389/fpubh.2022.1068253

**Published:** 2022-12-01

**Authors:** Shihui Shen, Xiaoyan Yuan, Jian Wang, Linfeng Fan, Junjun Zhao, Jiang Tao

**Affiliations:** ^1^Department of General Dentistry, Shanghai Ninth People's Hospital, Shanghai Jiao Tong University School of Medicine, Shanghai, China; ^2^College of Stomatology, Shanghai Jiao Tong University, Shanghai, China; ^3^National Center for Stomatology, Shanghai, China; ^4^National Clinical Research Center for Oral Diseases, Shanghai, China; ^5^Shanghai Key Laboratory of Stomatology, Shanghai, China; ^6^Shanghai Research Institute of Stomatology, Shanghai, China; ^7^Department of Radiology, Shanghai Ninth People's Hospital, Shanghai Jiao Tong University School of Medicine, Shanghai, China

**Keywords:** dental age, development, machine learning, youth, panoramic

## Abstract

**Background:**

Machine learning (ML) algorithms play a key role in estimating dental age. In this study, three ML models were used for dental age estimation, based on different preprocessing methods.

**Aim:**

The seven mandibular teeth on the digital panorama were measured and evaluated according to the Cameriere and the Demirjian method, respectively. Correlation data were used for decision tree (DT), Bayesian ridge regression (BRR), k-nearest neighbors (KNN) models for dental age estimation. An accuracy comparison was made among different methods.

**Subjects and methods:**

We analyzed 748 orthopantomographs (392 males and 356 females) from eastern China between the age of 5 and 13 years in this retrospective study. Three models, DT, BRR, and KNN, were used to estimate the dental age. The data in ML is obtained according to the Cameriere method and the Demirjian method. Coefficient of determination (R^2^), mean error (ME), root mean square error (RMSE), mean square error (MSE) and mean absolute error (MAE), the above five metrics were used to evaluate the accuracy of age estimation.

**Results:**

Our experimental results showed that the prediction accuracy of dental age was affected by ML algorithms. MD, MAD, MSE, RMSE of the dental age predicted by ML were significantly decreased. Among all the methods, the KNN model based on the Cameriere method had the highest accuracy (ME = 0.015, MAE = 0.473, MSE = 0.340, RMSE = 0.583, R^2^ = 0.94).

**Conclusion:**

The results show that the prediction accuracy of dental age is influenced by ML algorithms and preprocessing method. The KNN model based on the Cameriere method was able to infer dental age more accurately in a clinical setting.

## Introduction

Age estimation through the application of dental morphology and radiology plays an important role in clinical medicine and forensic medicine (1–3). Age can now be estimated by skeletal maturation or dental development (4, 5). However, age estimation may be biased when individuals encounter chronic diseases or nutritional deficiencies during growth and development. Compared to skeletal maturity, dental development is less influenced by the environment, which may be related to the strict genetic control of tooth development (6, 7).

The Demirjian method classifies the teeth into eight stages from A to H depending on maturity and calcification (8). The sum of seven permanent teeth in the left lower jaw corresponds to different ages for boys and girls, respectively. The Demirjian method relies on the subjective judgment of the assessor involved, which may lead to a high level of error. Recently, a new method was invented by Cameriere, which has been widely used all over the world (9). It is a European formula that measures the open apices of the seven permanent teeth in the left lower jaw by means of panoramic radiographs. This method is more objective by measuring data related to the teeth to estimate the dental age.

Both of these methods have been applied in China. The study by Ye et al. (10) showed that the dental age estimated with the Demirjian method was 1.68 years higher than the chronological age for males and 1.28 years higher for females. Shen et al. showed that the dental age obtained with the European formula was underestimated by 0.690 years for males and 0.484 years for females (11). However, by using machine learning (random forest, support vector machine, and linear regression model), the difference between dental age and chronological age was reduced to < 0.01 years.

Machine learning (ML) methods are widely used for early prediction and identification of different types of diseases (12–14). We are very pleased to see that ML is gradually being used in clinical medicine, and that the combination of medicine and engineering is opening up more possibilities for clinical practice. Brain age, ophthalmology and dental age have all been studied with ML (12, 13, 15). For adolescent children, the mean absolute error (MAE) for age estimation using ML was < 1 year in all cases (11, 13, 16). In 2021, Galibourg et al. (16) reported surprising results for machine learning based on the Demirjian method for estimating age without using traditional conversion tables. For adults, the MAE was 6.022 years when dental age was estimated (17).

The advent of ML has further reduced the error in age estimation and made age estimation more reliable and practical. Many studies have applied ML to the estimation of bone age (18), and the application of ML to dental age is gradually increasing (11, 16). Studies have shown that ML is more accurate than traditional radiological methods. With ML, the accuracy of both methods has been improved (11, 16). However, it has not been discussed about which is more accurate when both Demirjian and Cameriere methods use ML.

The purpose of this study is to compare (1) the dental age prediction ability of Demirjian and Cameriere methods by ML algorithm (2) the dental age prediction ability of 3 ML algorithms: decision tree (DT), Bayesian ridge regression (BRR), k-nearest neighbors (KNN) model.

## Materials and methods

### Samples

The research was authorized by the Independent Ethics Committee of the Shanghai Ninth Hospital affiliated with Shanghai Jiao Tong University, School of Medicine (2017-282-T212). All methods were performed in accordance with relevant guidelines and regulations. Informed consent was obtained from all subjects or their legal guardian(s) participating in the study.

This retrospective study selected digital panoramic radiographs taken by KODAK 8000C Panoramic and Cephalometric Digital Dental X-ray Machine collected during outpatient treatment between 2000 and 2013. A total of 748 panoramic images of adolescents aged 5–13, including 356 females and 392 males, were included in this study. Their age and gender distributions are shown in Table 1. Since the date of birth and the date of taking the panoramic images were known for each subject, the chronological age was calculated as the difference between these two dates, rounded to two decimal places.

**Table 1:** Age groups and gender distribution.

**Age group**	**Gender**		**Total**
	**Female**	**Male**	
5.00–5.99	20	18	38
6.00–6.99	47	45	92
7.00–7.99	35	44	79
8.00–8.99	52	42	94
9.00–9.99	48	63	111
10.00–10.99	44	48	92
11.00–11.99	45	43	88
12.00–12.99	35	45	80
13.00–13.99	30	44	74
Total	355	392	748

The inclusion criteria for panoramic radiographs are as follows: complete mandibular permanent teeth (except third molars); clearly visible root development; no systemic disease; no history of root canal therapy; no related diseases affecting mandibular development, such as cysts or cancer.

### Dental age estimation

Digital panoramic radiographs were stored on a computer and processed by computer-aided measuring software (Adobe Photoshop CC 2017). All panoramic slices were pre-processed according to the Cameriere and Demirjian methods, and the relevant data were recorded.

**Figure 1:**
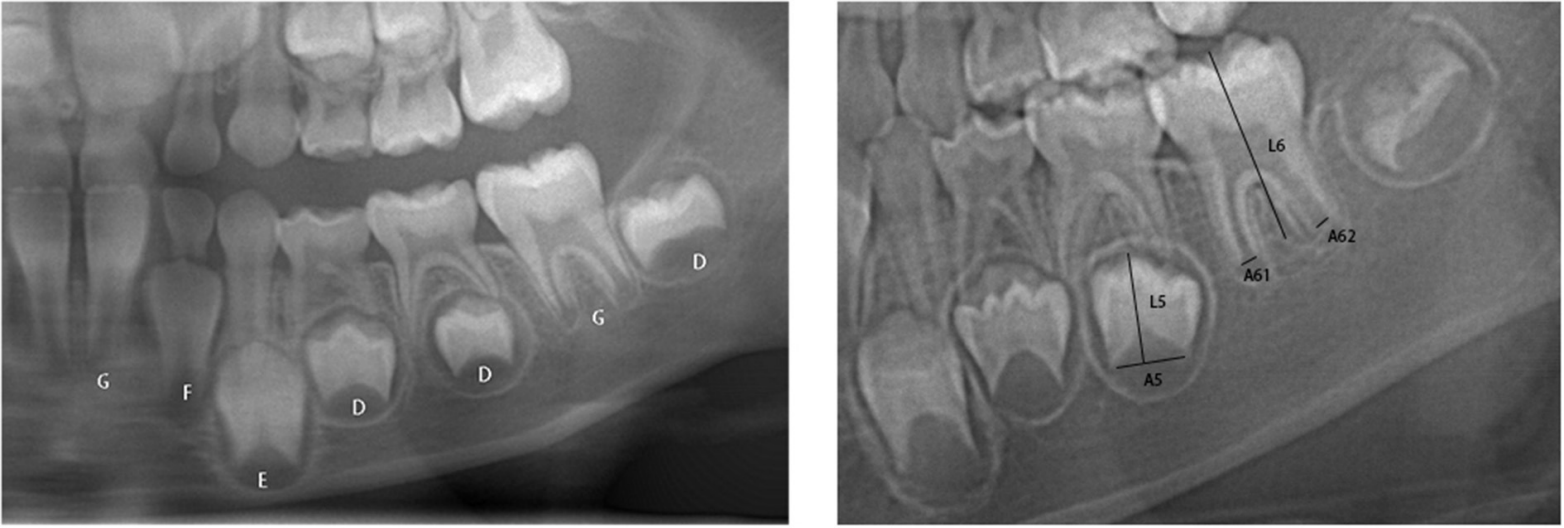
**(Left)** Schematic representation of the developmental assessment of seven molars according to the Demirjian method (the letters represent the stages of the Demirjian tooth age inference method). **(Right)** Schematic representation of the measurement of single-rooted and double-rooted teeth according[[Inline Image]] to the Cameriere method.

According to the Demirjian method, 7 mandibular teeth were classified into eight stages from A to H according to their development and mineralization (8) (Figure 1, left). When it comes to Demirjian method, the assessment grade of the7 permanent teeth and the gender of the sample were included into the ML algorithm as variables. In the Demirjian method, we used one-hot encoding. One-Hot encoding, also known as one-bit valid encoding, essentially encodes N states using N-bit state registers, each with its own register bits, with only one bit valid at any time.

The Cameriere method, in short, divides the distance (A_i_, i = 1, ..., 7) between the inner sides of the open apex of each of the seven left mandibular teeth by the length of the tooth (L_i_, i = 1, ...,7) to obtain the normalized value (X_i_ = Ai/ Li, i = 1, ...,7). Xi (i = 1, …, 7) = 0 if tooth development is complete and the apical foramen is closed (Figure 1, right) (9). The standard values (X_i_, i = 1, ..., 7) of the seven permanent teeth and the gender of the sample were included into the ML algorithm as variables, when it comes to Cameriere method.

The following ML supervised regression algorithms were tested, DT, BRR and KNN model. Out-of-sample performance have been assessed by means of the well-known K-fold cross-validation. More precisely, in this study, we divided the data into ten groups. The image data were divided into 10 groups, 9 groups are used as training data and one for validation (19, 20). This process was repeated 10 times until each of these 10 sets became the validation dataset. The machine learning models based on Demirjian method were trained on the information sources as follows: gender (g) and the assessment grade of the seven permanent teeth. The machine learning models based on Cameriere method were trained on the information sources as follows: gender (g), the normalized measurements of the seven permanent developing teeth on the left mandible (X_i_, i = 1, …,7), the sum of the normalized open apices (s, s = X_1_+ X_2_+…+ X_7_), the number of teeth with complete root development (N_0_) and the first-order relationship between s and N_0_ (s·N_0_). The target value was the chronological age.

All three ML algorithms are supervised learning. DT is a supervised learning methodology commonly adopted for both classification and regression (21). Ridge regression is a model tuning method that is used to analyze the data that suffers from multi-collinearity (22). Ridge regression treats the model parameters as fixed and finds estimates by minimizing a penalized cost function. BRR is the Bayesian counterpart, hence, in this framework the parameters are random variables whose prior distribution, exploiting Bayes rule, is updated by the data likelihood, leading to a posterior distribution for the parameter vector. It must be pointed out that the Bayesian framework allows a more natural uncertainty quantification, since it allows for an explicit probabilistic interpretation to parameters.

The KNN algorithm is supervised learning, where each piece of data in the training sample set has a definite known label. KNN does not have an explicit learning process and is, in fact, a well-known representative of lazy learning. The KNN algorithm itself is simple, easy to understand and implement, and it is highly accurate and insensitive to outliers (23, 24). The disadvantages are long computation time, high complexity and high memory overhead, as the training data needs to be parked in memory.

To avoid overfitting, a 20% validation dataset was used during hyperparameter optimization. The tuning of the hyperparameters to obtain the best model was achieved by exploring multiple combinations using the GridSearchCV function. The hyperparameters described in Supplementary Table S1 were tuned.

The method of age inference may underestimate or overestimate age, which is known as prediction error. An accurate method has low prediction error according to some performance metric, meaning that the average difference between dental age and chronological age will be close to zero. Accuracy refers to how large the difference between the dental age (DA) and chronological age (CA). The difference between DA and CA can be expressed in many ways according to the considered performance metric, such as mean absolute error, median absolute error, etc.

The chronological age of each subject was calculated by subtracting the date of birth from the date the panoramic was taken. In this study, the mean error (ME) of dental age and chronological age was calculated to quantify the direction of error (CA-DA), where positive values indicate that dental age is underestimated and negative values conversely. The mean absolute difference (MAD) of dental age and chronological age was calculated to quantify the magnitude of the error. In addition to the above two metrics, three other indicators were used to asses accuracy, the coefficient of determination (R^2^), root mean square error (RMSE) and mean square error (MSE) were also used to evaluate the accuracy of age estimation.

Data analysis and related icon production were performed through SPSS 25.0 (IBM Corp. Released 2017. IBM SPSS Statistics for Windows, Version 25.0. Armonk, NY: IBM Corp.), Pycharm 2021 and Python 3.8.2. The significance level was set at 5%.

### Intra and interobserver agreement

Since the possibility of reliably replication measurements is an important component of any measurement study, both intra- and interobserver error was tested. Both observers participating in the study were trained in age estimation methods. Each observer assessed each of the 748 radiographs. To assess interobserver reliability, both observers twice evaluated 50 randomly selected X-rays before starting the original study. The intraobserver correlation coefficient (ICC) for the intra-observer agreement was 0.92 for both observers, whereas it was 0.86(for Cameriere method) and 0.82(for Deimirjian method) for the inter-observer agreement. The results of the intra-class correlation coefficient show the inter-observer and test-recovery reliability.

## Results

A total of 748 panoramic images, 356 females and 392 males, were included in this study. The distribution of males and females in each group was relatively even. The performance metrics are shown in Table 2.

**Table 2:** Mean error (ME), mean absolute error (MAE), mean square error (MSE), root mean square error (RMSE), and R^2^ values assessing performance of machine learning regression methods based on Demirjian and Cameriere method, respectively.

**Method**	**ML model**	**ME**	**MAE**	**MSE**	**RMSE**	**R^2^**
Demirjian	Traditional	−0.647	0.982	22.254	4.717	–
	BRR	−0.002	0.510	0.404	0.636	0.928
	DT	0.011	0.523	0.609	0.780	0.892
	KNN	−0.027	0.517	0.435	0.660	0.923
Cameriere	Traditional	0.592	0.846	0.755	0.869	–
	BRR	−0.030	0.535	0.436	0.660	0.923
	DT	0.052	0.584	0.601	0.775	0.893
	KNN	−0.015	0.473	0.340	0.583	0.940

Compared with traditional methods, whether it is Deimirjian or Cameriere method, ME, MAE, MSE and RMSE have been greatly reduced after the optimization of ML, which means that the accuracy is greatly improved. When using the traditional Deimirjian method, its ME and MAE were −0.647 and 0.982, respectively, and the MSE and RMSE were 22.254 and 4.717. That is, the dental age estimated by the traditional Deimirjian method was 0.647 year higher than the chronological age, with an error of 0.982 year. With the aid of ML, the ME of the three models (BRR, DT, and KNN models) were −0.002, 0.011 and −0.027, respectively. The MAE also dropped to 0.510, 0.523, and 0.517. ML also had obvious advantages in the Cameriere method. Before using ML, the ME and MAE of the Cameriere method were 0.592 and 0.846, respectively. After optimization with ML, the ME became −0.030 (BRR), 0.052 (DT) and −0.015 (KNN), and the MAE dropped to 0.535 (BRR), 0.584 (DT) and 0.473 (KNN).

**Figure 2:**
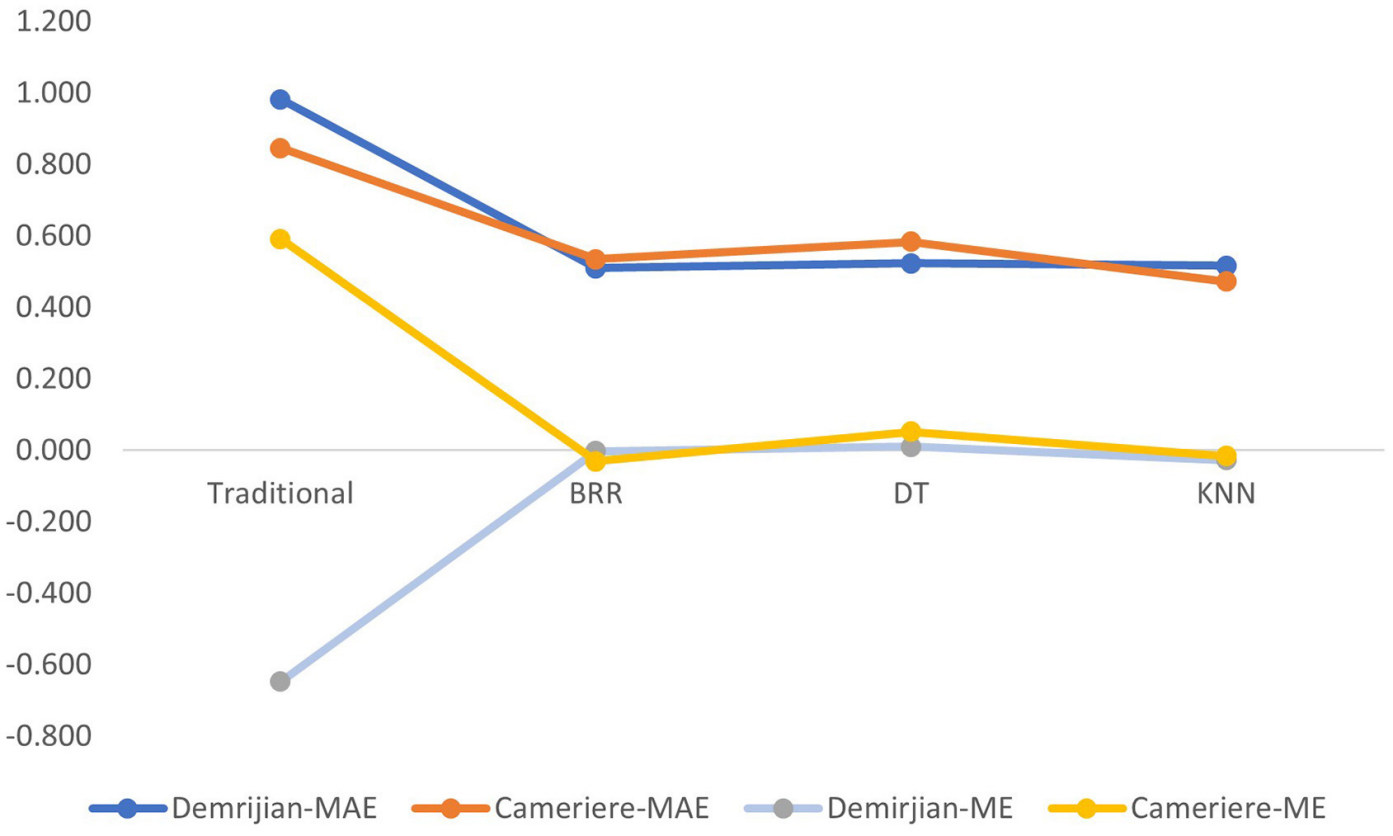
Mean error (ME) and mean absolute error (MAE) of different machine learning algorithms (DT, decision tree; BRR, Bayesian ridge regression; KNN, k-nearest neighbors) based on Demirjian or Cameriere method.

For the Cameriere method, among the three ML models, the KNN model performed the best, with the highest R^2^ (0.940) and the smallest ME and MAE. The DT model had the lowest R^2^ (0.893), that was, the lowest fit. In the Deimirjian method, the DT model was also a ML model with the lowest fitting degree (R^2^ = 0.892). The best performer in the Deimirjian method was the BRR model (R^2^ = 0.928, ME = −0.002, MAE = 0.510), followed by the KNN model (R^2^ = 0.923, ME = −0.027, MAE = 0.517) (Figure 2).

Among all the methods in this study, the smallest MAE is the KNN model ML based on the Cameriere method (MAE = 0.473), the dental age is only overestimated by 0.015 years, which is close to 0. The fit was also very high, at 0.94.

The linear equation was used to estimate the absolute error of the dependence of the sample population on the dental age under different methods (Table 3). Errors were only calculated for the test set during the 10-fold cross-validation. As show in this Table 3, the accuracy of the model decreases as individuals are getting older and ranges between 0.219 and 0.73 years.

**Table 3:** Typical absolute error, in dependency of dental age for different situations.

**Method**	**ML model**	**Dental age**								
		**5**	**6**	**7**	**8**	**9**	**10**	**11**	**12**	**13**
Demirjian	BRR	0.393	0.47	0.631	0.588	0.522	0.533	0.73	0.55	0.627
	DT	0.391	0.68	0.577	0.51	0.419	0.429	0.535	0.615	0.219
	KNN	0.32	0.498	0.634	0.506	0.475	0.558	0.549	0.595	0.347
Cameriere	BRR	0.425	0.474	0.538	0.455	0.448	0.482	0.673	0.555	–
	DT	0.318	0.382	0.462	0.472	0.426	0.462	0.575	0.617	0.298
	KNN	0.367	0.463	0.49	0.505	0.484	0.547	0.488	0.618	0.466

**Figure 3:**
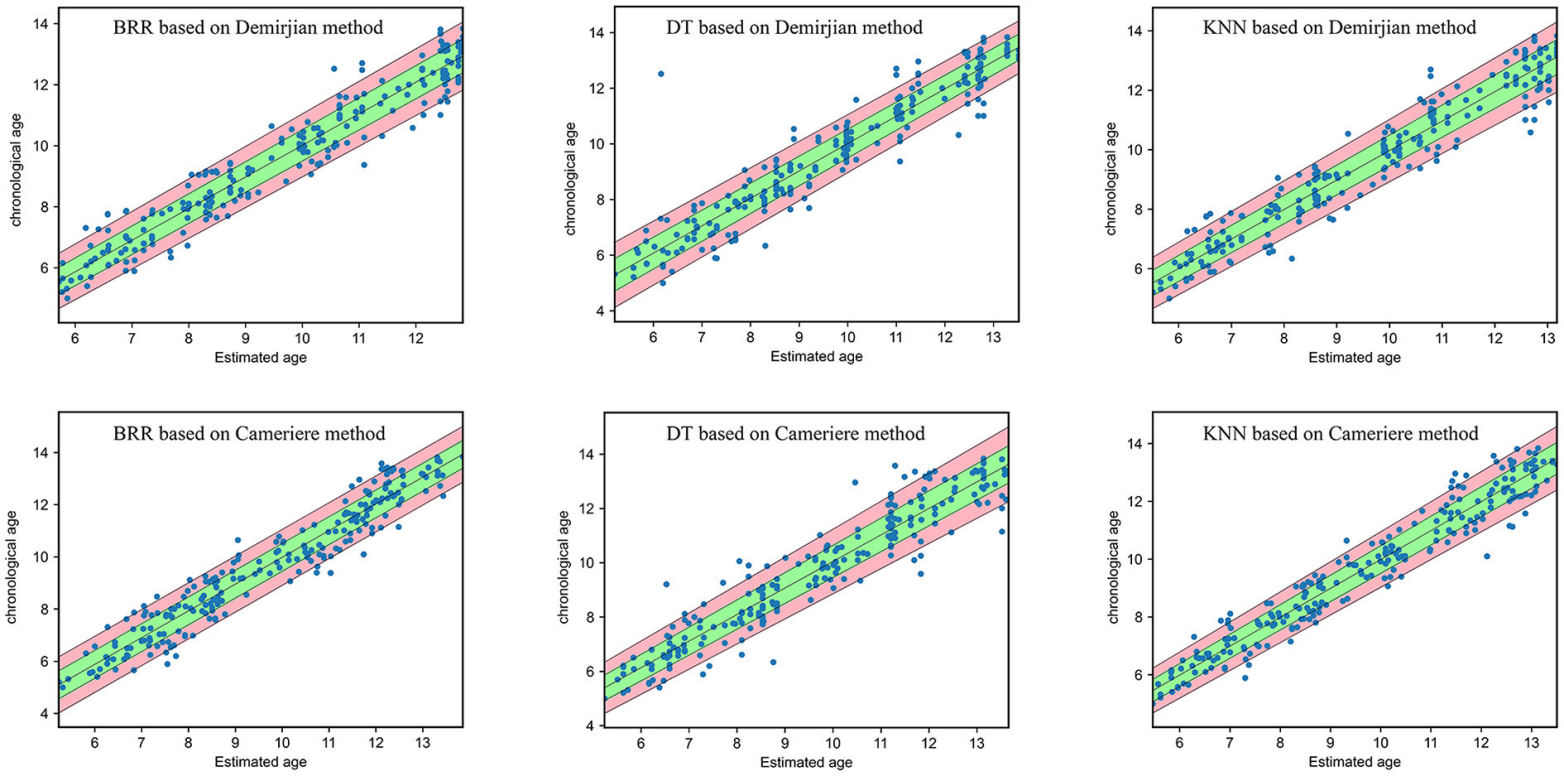
The model performance for different machine learning algorithms (DT, decision tree; BRR, Bayesian ridge regression; KNN, k-nearest neighbors) based on Demirjian or Cameriere method. Green area: dental age ±δ; red + green area: dental age ± 2δ; ideal line: chronological age = estimated dental.

As can be seen from Figure 3, most of the samples lie whithin the DA ± δ interval. For samples aged 5–13, the model was most accurate in the middle age group. Accuracy begins to decrease as the age of individuals age grows or decreases. The points are more concentrated under the KNN model. If the interval is doubled, i.e., DA ± 2**δ**, more individuals will be covered. In Figure 3, the green area corresponds to the interval DA ± **δ**, whereas the red area corresponds to the interval DA ± 2**δ**. The **δ** was approximated as linear function of absolute error in dependency of dental age. This means that the independent variable of **δ** was the dental age and the dependent variable was the corresponding absolute error.

**Figure 4:**
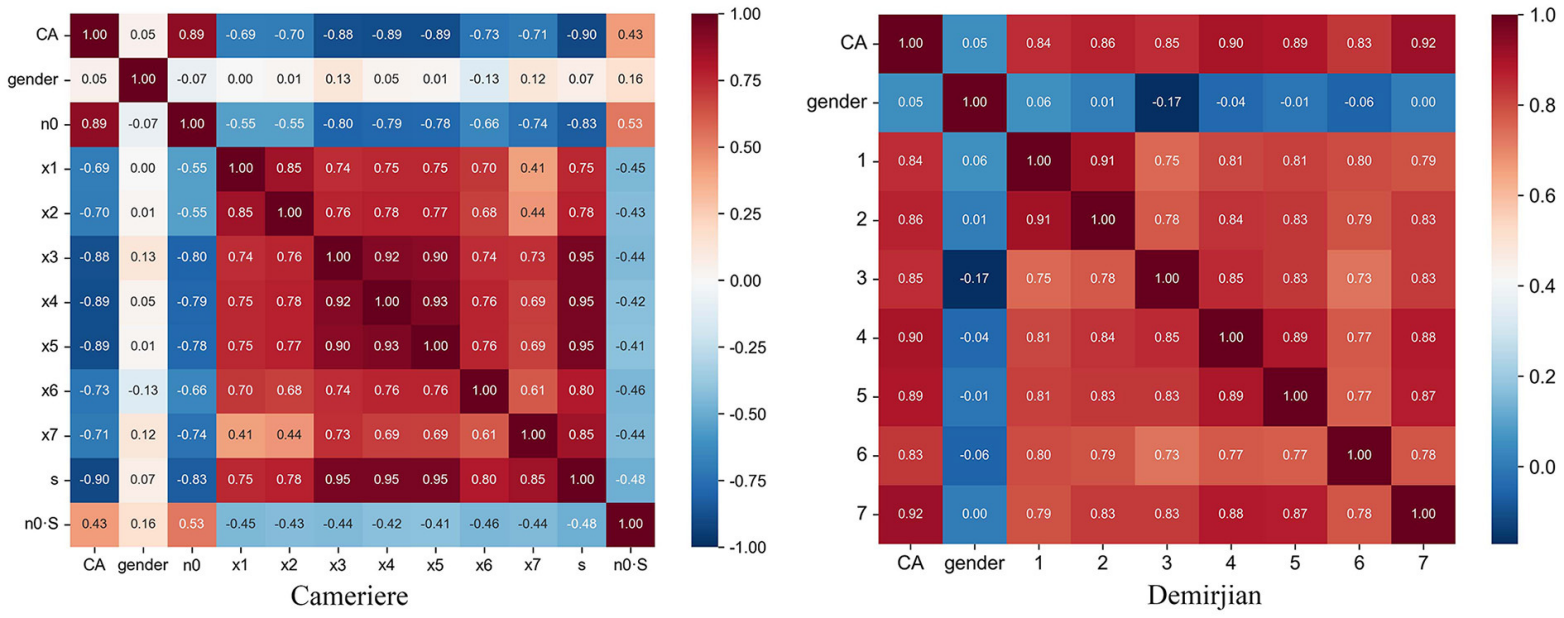
Heatmap of the correlation coefficient between the variables. **(Left)** The variables of the Cameriere method, gender (g), the normalized measurements of the seven permanent developing teeth on the left mandible (X_i_, i = 1, …,7), the sum of the normalized open apices (s, s = X_1_+ X_2_+…+ X_7_), the number of teeth with complete root development (N_0_) and the first-order relationship between s and N_0_ (s·N_0_). **(Right)** The variables of the Demirjian method, gender (g) and the assessment grade of the seven permanent teeth.

In addition, correlation coefficients were calculated for the respective variables of the Cameriere and Demirjian methods. The heat map of the correlation coefficients is shown in Figure 4. As seen in Figure 4, each variable has a correlation coefficient close to 1 or −1 with respect to the target value, meaning that they all contribute considerably to the prediction. The closer the correlation coefficient is to 1 or −1, the stronger the positive/inverse linear relationship, while a value of 0 indicates that there is no linear relationship between the two variables.

## Discussion

In oral forensics, the need to infer the age of life becomes increasingly important as more immigrants (illegal or otherwise) come to a country without a valid legal identity document. Second, missing or uncertain birth data is also common. For these reasons, more and more studies have been conducted on the estimation of dental age.

**Figure 5:**
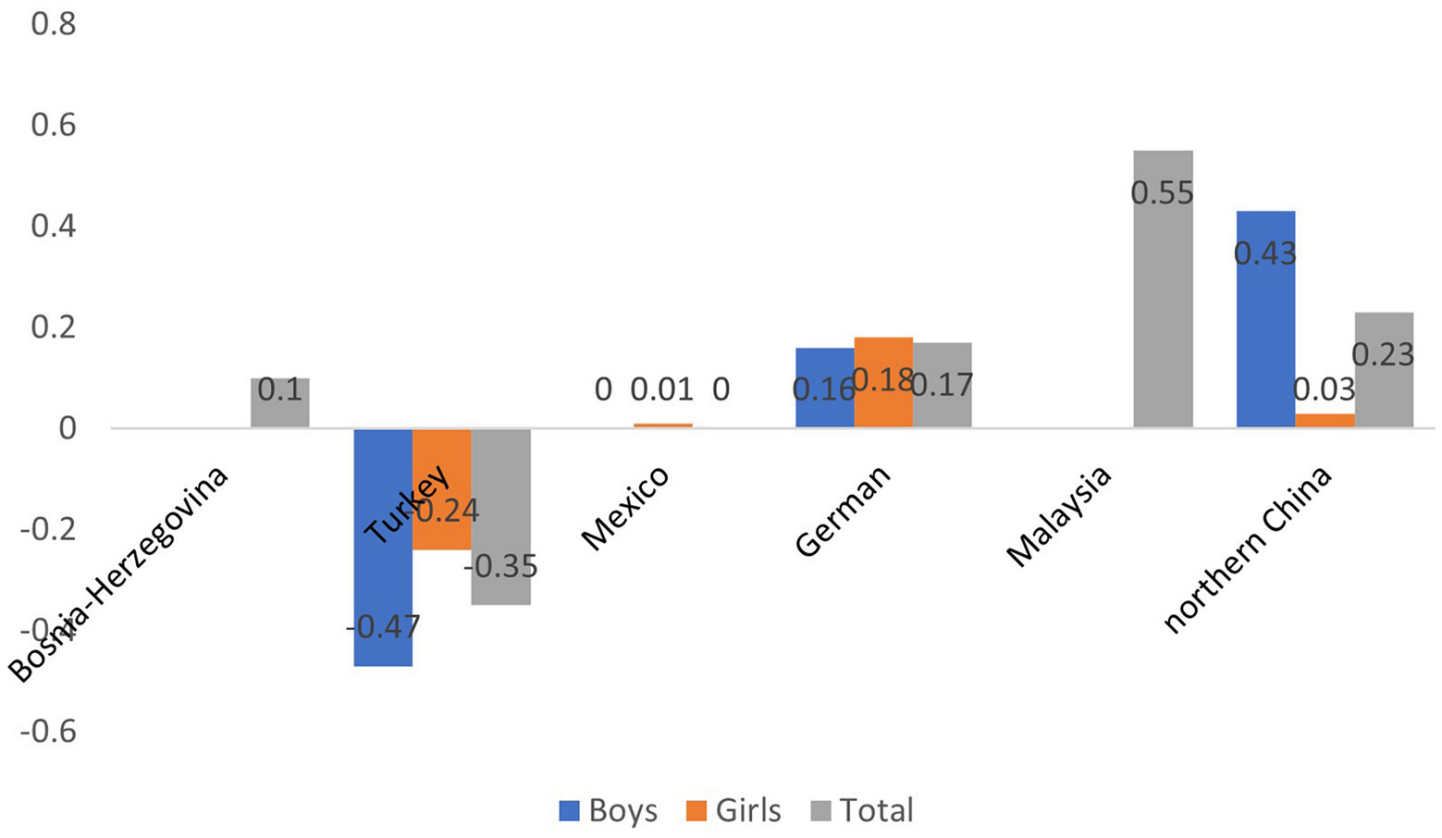
Application of Cameriere European formula in various regions.

In other studies on the Cameriere's European formula, children in Bosnia-Herzegovina overestimated the dental age of girls by 0.10 year and underestimated the dental age of boys by 0.02 year (25); the dental age in Turkey was underestimated by 0.35 year (0.24 year for girls and 0.47 year for boys) (26, 27); the mean dental age of girls in Mexico was overestimated by 0.01 year, while the age of boys was underestimated by 0.00 year (28). In addition, in Germany, the dental age was overestimated by 0.16 years for boys and 0.18 year for girls (29); Malaysian Chinese children were overestimated by 0.50 year (0.33 year for girls and 0.66 year for boys) (30). Among children in northern China, the dental age was overestimated by 0.23 year (0.43 year for boys and 0.03 year for girls) (31). This suggests that Cameriere's European formula is more accurate in inferring the age of European children (Figure 5), whereas it is less accurate in other populations, which may be related to ethnicity.

Take a look at Demirjian in use around the world. A study by Jayaraman et al. (32) showed that the dental age was overestimated by 0.60 year in French–Canadian male and 0.65 year in females. According to Maber et al. (33) the Demirjian method was found to overestimate age in Bengalis and British Caucasians with an average accuracy of 0.25 years in males and 0.23 years in females. Previously, Wang et al. (34) investigated the applicability of the Demirjian method in adolescents in eastern China and found that the dental age inferred with these methods was underestimated. The findings were consistent even when the subjects were in different age groups (6–13 years in this study and 11–18 in Wang et al.'s study). Both Cameriere and Demirjian seem to be geographically limited in their application.

A study by Gaur et al. (35) showed that children born in families of higher socioeconomic status develop teeth earlier than children from low-income families. Hiernaux et al. (36) found that three-quarters of African-American have teeth that erupt earlier than Caucasians in Europe or the United States. According to Olze et al. (37), the average age of third molar eruption is 0.5–3 years and 0.5–2 years higher in Asians and Africans than in Caucasians. Thus, environmental and ethnic differences, as well as genetic factors, may contribute to these differences.

The inclusion of ML can substantially improve the accuracy of age inference. Among the three ML algorithms in this study, the DT model performed the worst. Perhaps this is because age estimation is a regression problem rather than a binary classification problem. Therefore, regression models (such as KNN in this study) are more suitable for age estimation. In the study of Galibourg et al. (16), KNN also has good performance (ME = 0.009, MAE = 0.738) in the ML algorithm based on Demirjian method. This is consistent with our findings.

Convolutional neural network (CNN) is part of deep learning, a new research direction in machine learning that is more complex and specialized. A study by Zaborowicz et al. (38) showed that the MAE for dental age inference using CNN in children aged 4–15 years ranged from 0.195 to 0.384 years. The MAE for the current study ranged from 0.21 to 0. 73 years. Compared to traditional manual methods, both deep learning and machine learning regression methods showed high accuracy with mean errors close to zero.

This is the first comparison of the accuracy of the Cameriere and Demirjian methods of dental age estimation using ML simultaneously. The ML demonstrated its excellent performance, with very low MAE, ME MSE and RMSE in dental age. We believe that this breakthrough will significantly increase the feasibility of clinical dental age estimation. ML algorithm helps to establish assessment criteria and improve the accuracy of dental age estimates in local populations.

The present study provides a reference for future clinical applications of dental age, such as the assessment of child development and the projection of the age of the missing population. The sample for this experiment was drawn from hospitals in eastern China, filling a gap in machine learning for dental age inference in that region. This further increases the potential for global clinical application of dental age. Of course, this study still has geographical limitations, the data from other regions were not collected, which needs to be further confirmed in subsequent experiments. Individual differences caused by developmental differences and nutritional dietary habits of different ethnic groups are inevitable in the extrapolation of dental age. Perhaps including samples from more regions in the ML could reduce such errors. In addition, further research is needed to develop models regarding fully automated dental age inference. We believe that as the sample size increases, the accuracy of machine learning will further improve.

## Conclusion

In this study, ML was shown to greatly improve the accuracy of dental age inference. It was shown that the accuracy of dental age inference was higher than that of the traditional Demirjian method or Cameriere European formula, for both KNN, DT and BRR models. The highest accuracy among the machine learning dental age inference based on preprocessing of different methods was the KNN model based on the Cameriere method (ME = 0.015, MAE = 0.473, MSE = 0.340, RMSE = 0.583, R^2^ = 0.94).

In future studies, ML can be used for dental age estimation in a larger geographical area and over a larger age range.

## Data availability statement

The original contributions presented in the study are included in the article/Supplementary material, further inquiries can be directed to the corresponding authors.

## Ethics statement

The studies involving human participants were reviewed and approved by the Independent Ethics Committee of the Shanghai Ninth Hospital affiliated to Shanghai Jiao Tong University, School of Medicine (2017-282-T212). Written informed consent to participate in this study was provided by the participants' legal guardian/next of kin.

## Author contributions

SS: methodology and writing original draft. XY: investigation and resources. JW and LF: methodology and formal analysis. JZ and JT: conceptualization, supervision, and project administration. All authors have read and approved the final manuscript.

## Funding

This work was sponsored by the Interdisciplinary Program of Shanghai Jiao Tong University (YG2019ZDA07), Innovative Research Team of High-Level Local Universities in Shanghai (SHSMU-ZLCX20212401), and Shanghai Sailing Program (No. 21YF1424100).

## Conflict of interest

The authors declare that the research was conducted in the absence of any commercial or financial relationships that could be construed as a potential conflict of interest.

## Publisher's note

All claims expressed in this article are solely those of the authors and do not necessarily represent those of their affiliated organizations, or those of the publisher, the editors and the reviewers. Any product that may be evaluated in this article, or claim that may be made by its manufacturer, is not guaranteed or endorsed by the publisher.
