# Supplementary material for: Evaluation of a machine learning algorithms for predicting the dental age of adolescent based on different preprocessing methods
Source: Front Public Health. 2022 Dec 1;10:1068253. doi: 10.3389/fpubh.2022.1068253 (PMC9751184; doi:10.3389/fpubh.2022.1068253)
Supplement: Supplementary file 1 [file Table_1.docx]

Supplementary Table 1: List of the tuned hyperparameters for each Machine Learning algorithm. For each hyperparameter, the values inside square brackets were explored by Grid Search.

| K-Nearest Neighbors (KNN) | *n_neighbors*: the number of neighbors or K to use [5 to 13] *weights*: the weight function used in prediction [uniform or distance]  *algorithm*: type of algorithm used to compute the nearest neighbors [ball tree, kd tree or brute] |
| --- | --- |
| Decision Tree (DT) | *criterion*: the function to measure the quality of a split in the tree [mse, Friedman mse or mae]  *max_depth*: the maximum depth of the tree [6 to 8]  *max_features*: the number of features to consider when looking for the best split at a node [auto, sqrt, log2]  *min_samples_split*: the minimum number of samples required to split an internal node [2 to 5]  *min_samples_leaf:* the minimum number of samples required to be at a leaf node [2 to 5]  *splitter*: the strategy used to choose the split at each node [best or random] |
| Bayesian Ridge Regression (BRR) | *alpha_1*: shape parameter for the Gamma distribution prior over the alpha parameter [1e^-15^, 1e^-10^, 1e^-8^, 1e^-4^, 1e^-3^, 1e^-2^, 1, 5, 10, 20] *alpha_2*: inverse scale parameter (rate parameter) for the Gamma distribution prior over the alpha parameter [1e^-15^, 1e^-10^, 1e^-8^, 1e^-4^, 1e^-3^, 1e^-2^, 1, 5, 10, 20] *lambda_1*: shape parameter for the Gamma distribution prior over the lambda parameter [1e^-15^, 1e^-10^, 1e^-8^, 1e^-4^, 1e^-3^, 1e^-2^, 1, 5, 10, 20] *lambda_2*: inverse scale parameter (rate parameter) for the Gamma distribution prior over the lambda parameter [1e^-15^, 1e^-10^, 1e^-8^, 1e^-4^, 1e^-3^, 1e^-2^, 1, 5, 10, 20] |
